# Molecular Docking Simulations on Histone Deacetylases (HDAC)-1 and -2 to Investigate the Flavone Binding

**DOI:** 10.3390/biomedicines8120568

**Published:** 2020-12-04

**Authors:** Bernardina Scafuri, Paola Bontempo, Lucia Altucci, Luigi De Masi, Angelo Facchiano

**Affiliations:** 1 Department of Chemistry and Biology “A. Zambelli”, University of Salerno, Via Giovanni Paolo II, 132, Fisciano, 84084 Salerno, Italy; bscafuri@unisa.it; 2 Department of Precision Medicine, University of Campania “Luigi Vanvitelli”, via L. De Crecchio 7, 80138 Naples, Italy; paola.bontempo@unicampania.it (P.B.); lucia.altucci@unicampania.it (L.A.); 3 National Research Council (CNR), Institute of Biosciences and BioResources (IBBR), via Università 133, Portici, 80055 Naples, Italy; 4 National Research Council (CNR), Institute of Food Science (ISA), via Roma 64, 83100 Avellino, Italy

**Keywords:** epigenetics, histone deacetylase inhibitors, flavones, molecular simulations

## Abstract

Histone modifications through acetylation are fundamental for remodelling chromatin and consequently activating gene expression. The imbalance between acetylation and deacetylation activity causes transcriptional dysregulation associated with several disorders. Flavones, small molecules of plant origin, are known to interfere with class I histone deacetylase (HDAC) enzymes and to enhance acetylation, restoring cell homeostasis. To investigate the possible physical interactions of flavones on human HDAC1 and 2, we carried out in silico molecular docking simulations. Our data have revealed how flavone, and other two flavones previously investigated, i.e., apigenin and luteolin, can interact as ligands with HDAC1 and 2 at the active site binding pocket. Regulation of HDAC activity by dietary flavones could have important implications in developing epigenetic therapy to regulate the cell gene expression.

## 1. Introduction

The fine remodelling of the chromatin structure by post-translational covalent modifications (acetylation, methylation, phosphorylation, and clipping) of histone tails is a key mechanism for epigenetic regulation of gene expression [1,2]. In this context, histone acetyltransferases (EC 2.3.1.48, HAT) and histone deacetylases (EC 3.5.1.98, HDAC) are essential enzymes in adding and removing, respectively, the acetyl moiety on the amino acid lysine [1]. N-terminal tails of histones deacetylated by HDAC have positive charges that interact with the negatively charged phosphate groups of DNA. Consequently, the chromatin is condensed into a compact structure (heterochromatin) associated with low levels of gene transcription. This structural condition can be reversed by HAT activity to a relaxed and transcriptionally active DNA (euchromatin). Therefore, the levels of histone acetylation are the result of the HAT/HDAC activity balance that plays a crucial role in the regulation of gene transcription through modulation of epigenetic changes. Alterations of this tightly coordinated molecular system have been implicated in a range of diseases including inflammation, cardiovascular and neurodegenerative disorders, diabetes, and cancer [3,4].

To date, 18 eukaryotic HDAC are known and grouped into four classes on the basis of their structural and catalytic similarity [3]. Class I with HDAC1, 2, 3, and 8 is subdivided into the three subclasses Ia (HDAC1 and 2), Ib (HDAC3), and Ic (HDAC8). Class II is formed by the two subclasses IIa (HDAC4, 5, 7, 9) and IIb (HDAC6 and 10). Class III consists of NAD^+^ dependent HDAC homologous to the yeast Sir2 protein (Sir2-like or sirtuins: Sirt1-7). Class IV only includes HDAC11. The common characteristic to classes I, II, and IV is a catalytic domain with one histidine (His) and two aspartate (Asp) residues associated with Zn^2+^ ion cofactor responsible for Zn^2+^-dependent hydrolysis of ε-N-acetylated lysine residues to yield deacetylated histone by a charge-relay mechanism [5]. Overall, HDACs have a conserved domain belonging to the open alpha/beta fold class. This central core consists of alpha-helices alternating with parallel beta-strands that build a central beta-sheet. From here, large loops associate to form a binding pocket where, in depth, the Zn^2+^ ion participates in the enzyme active site.

**Figure 1:**
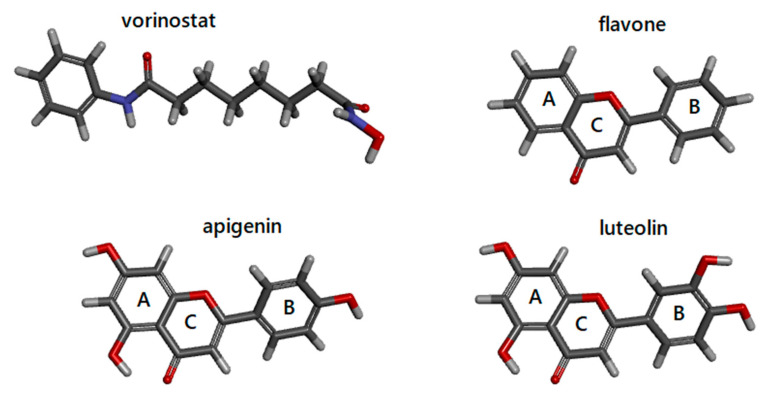
Molecular structures of vorinostat, flavone, apigenin, and luteolin are from the PubChem database. A, B, and C indicate the different rings of flavones. Images are generated by DiscoveryStudio4.5.

HDAC activity can be regulated at different levels by transcriptional regulation, post-translational modifications, subcellular localization, protein–protein interactions, proteolytic regulation, and small-molecules acting as HDAC inhibitors (HDACi). Thus, HDAC are attractive targets for the development of novel drugs, and HDACi may constitute potential therapeutic agents. Based on their structural characteristics, HDACi of natural origin are subdivided in hydroxamates, benzamides, cyclic peptides, and short-chain fatty acids [2]. Among the inhibitors acting on classes I-II HDACs, the anticancer agents Vorinostat (suberoylanilide hydroxamic acid or SAHA, Figure 1) and the structurally related Trichostatin A (TSA) exert multiple biological effects by interfering with the cell cycle, inducing apoptosis, autophagy, oxidative stress, and inhibiting angiogenesis [6,7]. SAHA and TSA reversibly bind to the HDAC active site, where chelate the cofactor Zn^2+^ by their hydroxamic acid group. NAD^+^ dependent HDAC belonging to class III (sirtuins) are not inhibited by conventional HDACi such as TSA and SAHA [8].

Since clinically used HDACi still experience adverse effects, to identify novel, more potent and specific inhibitors, plant-derived compounds have been screened and tested against human diseases, including cancers, for their ability to restore gene expression alterations [2,6,9,10]. A previous study on the acetonic extract from fruits of *Feijoa sellowiana* O. Berg has identified the bioactive component 2-phenyl-1,4-benzopyrone (known as flavone) to show anti-cancer action on solid and haematological cancer cells via HDAC inhibition [9]. This natural inhibitor, together with apigenin and luteolin, belongs to the subclass of flavonoids called flavones and is structurally different from known HDACi. Flavonoids have a backbone of 15 carbon atoms formed by two phenyl rings (A and B) and a heterocyclic ring with oxygen (C) abbreviated C6-C3-C6 (Figure 1). They are characterized by a double bond in position 2–3 and a ketone in position 4 of the ring C. Luteolin and apigenin have additional hydroxyl groups on the A and B rings. Flavonoids are chemopreventive molecules, ubiquitously present in different plant organs [2,10,11,12]. However, the exact action mechanism at the molecular level of inhibitory effects is still not fully understood [9].

The observation that the apoptotic activities of flavones are correlated with the same targets of HDACi [9,13,14] inspired us to carry out an in silico molecular characterization of underlying inhibition mechanism on HDAC of flavone and its derivatives apigenin and luteolin. In fact, the activity of these flavones has been already observed by experimental results reported in the literature [9,13,14], but it is not known how these molecules bind and inhibit their protein targets. We investigated their possible interactions on subclass Ia HDAC1 and 2 in comparison with the better known HDACi vorinostat. In more detail, we used docking simulations to check the suitability of the three molecules to mimic the interaction occurring between HDAC2 and vorinostat, described by the structural model obtained by X-ray diffraction studies [1]. We simulated by molecular docking the ligand-protein interaction, showing that the flavonic ligands can bind HDAC1 and 2 at the active site, as the vorinostat does with HDAC2. Consequently, HDAC activity could be directly regulated by dietary flavones with important implications on global gene expression regulation.

## 2. Materials and Methods

The structures of human HDAC1 and HDAC2 were selected from RCSB PDB [15], file code 4BKX and 4LXZ, respectively. The HDAC2 structure 4LXZ is complexed with vorinostat, an hydroxamic acid that inhibits HDACs. By selecting this structure, we have an experimental reference of HDAC2-inhibitor binding for our study. We selected the highest-resolution structure available of the entire HDAC1, i.e., the structure 4BKX in complex with the dimeric ELM2-SANT domain of MTA1 from the NuRD complex, in the absence of an HDAC1 structure complexed with vorinostat; the dimeric domain has been removed to perform the docking simulation. Although the sequences of HDAC1 and HDAC2 are very similar and can be aligned with the shift of one position at the N-terminus and few gaps in the C-terminal portion (see Supplementary Figure S1), we used the amino acid numbers with a difference of 5 positions in agreement with the numbers in the PDB structures.

Docking simulations were performed between the two enzymes and vorinostat, flavone, apigenin, and luteolin, with AutoDock 4.2 and AutoDockTools4 [16], to verify the suitability of the molecular structure of flavone, apigenin, and luteolin to interact with HDAC1 and HDAC2 at the same binding site occupied by vorinostat. 

The crystallographic structure of HDAC2 with vorinostat from the PDB was used to perform a redocking test in order to check the correctness of the parameters used and to evaluate the binding energy. In this way, the value of the predicted binding energy was used to compare the docking results between HDAC1/2 and ligands.

The 3D structures of flavones were downloaded in .sdf format from the PubChem database (https://pubchem.ncbi.nlm.nih.gov) [17] and converted in .pdb format using UCSF Chimera (http://www.rbvi.uscf.edu/chimera) [18].

Two different docking approaches were performed: the blind docking, by setting a grid box to include the entire protein surface, and the focused docking, by setting the grid box only on the binding site of the protein, as reported by the PDB file annotations. For each docking simulation, we simulated three protein-ligand systems: (i) the protein structures without water molecules, as suggested by AutoDock 4.2 protocol; (ii) the protein structures with two water molecules in binding site; (iii) the ligand decorated with an ensemble of water molecules, which may then contribute to the interaction (hydration condition).

For all systems, the molecular docking was performed with flexible ligands and both by keeping the entire protein rigid and by making flexible the residues involved in the catalytic channel, selected by visual inspection of the structure and on the basis of AutoDock limits in a flexibility setting. For HDAC1, the flexible residues were His140, His141, Phe205, Asp264, Tyr303, and additionally Asp176 for apigenin and luteolin. For HDAC2, the flexible residues were His145, His146, Asp181, Phe210, Asp269, Tyr308, and additionally Phe155 for flavone. According to docking simulation protocols already in use in our laboratory [19,20], the ligands and the proteins were prepared using AutoDockTools, by adding hydrogens and partial charges in agreement with Gasteiger. The dimensions of the grid box were set according to the protein’s dimension. The Lamarckian Genetic Algorithm was employed, setting 100 independent Genetic Algorithm runs for each ligand; the other parameters were kept at default values. Detailed settings are reported in Supplementary Table S1.

Cluster analysis was performed on the docked results using a root mean square deviation (RMSD) tolerance of 2 Å, and the initial coordinates of the ligand were used as the reference structure. Subsequently, we selected for each ligand the result with the best binding energy value. To further investigate the cluster population, we selected the best five results in terms of binding energy, and evaluated their mean value with standard deviation.

Analysis of the ligand–protein interaction has been performed with DiscoveryStudio4.5 (Biovia, San Diego, CA, USA), used also to generate the 2D schemes of ligand-protein binding and 3D molecular images.

## 3. Results and Discussion

### 3.1. Docking Simulations of Inhibitor Vorinostat Interaction with HDAC1 and HDAC2

**Figure 2:**
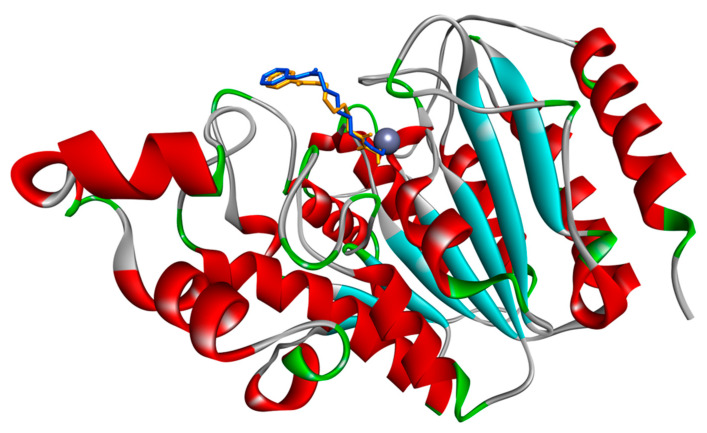
Interaction of the inhibitor vorinostat with HDAC2 from a crystallographic structure and re-docking simulations. The complex HDAC2-vorinostat obtained by redocking simulation has been superimposed to the crystallographic complex. Vorinostat from redocking (orange molecule in stick representation) occupies the same site as in the crystallographic complex (blue molecule). HDAC2 backbone is represented as a ribbon, with red helices and cyan strands. Grey sphere indicates the Zinc atom in the site. Image is generated by DiscoveryStudio4.5.

In this work, docking simulations were performed to explore the capability of flavone, luteolin, and apigenin to interact with HDAC1 and HDAC2 proteins. The availability of the crystal structure of HDAC2 in complex with the inhibitor vorinostat allowed us to use vorinostat as a reference point for settings of the docking procedure and comparison of binding energy values obtained with the natural compounds under study. Firstly, we re-docked vorinostat in the binding site of HDAC2. This procedure is useful to verify that the simulation protocol is able to reproduce an experimental proof. The result of the simulation showed that vorinostat is correctly positioned into the active site binding pocket of HDAC2 (see Figure 2) and gave favourable energy values of interaction (Table 1). The comparison of the vorinostat conformation from the experimental structure and from redocking procedure is also reported as a molecular superimposition image with RMSD in Supplementary Figure S2.

The simulation has been performed under different conditions (see Section 2) and the best interaction energy value obtained, i.e., −8.45 Kcal/mol (Table 1), referred to the focused docking in the presence of the crystallographic water molecules. Binding to the cofactor Zn^2+^ in HDAC2 is given by the hydroxamic acid group (Supplementary Figure S3), as expected [7]. Then, we also performed docking simulations between HDAC1 and vorinostat, and obtained a very similar interaction energy value, i.e., −8.46 Kcal/mol (Table 1). However, at the lowest energy the orientation of vorinostat into the HDAC1 catalytic site allows that the oxygen bound to C8 coordinates the cofactor Zn^2+^ (Supplementary Figure S4), different to HDAC2. By exploring the other conformations obtained in the docking simulations of vorinostat-HDAC1, the interaction of the hydroxamic acid group with the cofactor Zn^2+^ is also possible, though with higher energy, i.e., −6.71 Kcal/mol (data not shown), thus suggesting that two different binding modes may exist with HDAC1.

It is worth noting that, while the conformation at the lowest energy value represents the best interaction obtained, the docking simulations generate 100 conformations, clustered on the basis of their structural similarity. Therefore, we performed a deeper analysis of the results by comparing the best 3 conformations from the cluster containing the conformation with the lowest binding energy. The table with mean energy values and standard deviation (Supplementary Table S2) confirms the capability of flavone, luteoline, and apigenin to interact with HDAC1 and 2 with binding energy values similar to the value of vorinostat, or better.

### 3.2. Docking Simulations of Flavones Interaction with HDAC1 and HDAC2

To explore the possibility that flavone, luteolin, and apigenin bind to HDACs, we applied the same docking simulation protocol to HDAC1 and HDAC2 as target proteins and these three molecules as ligands, by testing different conditions. The best energy values obtained for each pair of protein-ligands are reported in Table 1.

Blind docking was performed to explore the entire surface of protein, searching for possible binding sites to be further investigated in more detail. In our case, the target active site of the protein is known, being already identified by crystallographic structure of HDAC2 in complex with the vorinostat inhibitor. However, in any case, the blind simulation is useful in finding alternative binding sites, as it helps to have a preliminary screening of the surface. Next, focused docking was performed on the preliminary sites resulting from the blind search, by setting a higher resolution screening. The blind docking identified in all cases the known active site pocket as the most reliable binding site for the ligands. The energy values of blind docking are commonly considered not optimized, being performed by searching grids with low definition. It is interesting to note that the blind docking suggested that flavone and apigenin might interact with better energy values than vorinostat, in both HDAC1 and HDAC2 (Table 1, higher part). On the contrary, the blind docking conditions suggested that luteolin might interact with HDAC1 in a worse way than vorinostat.

The results of blind docking have been further investigated by focusing on the binding site of HDACs. The results are shown in Table 1 (lower part). As expected, in all cases interaction energy values obtained with focused docking are lower than in blind docking, because the higher definition of the simulation allows to optimize the docking and find protein–ligand interactions with lower energies. Previous docking simulations reported better docking scores for givinostat, another known inhibitor of HDACs, than apigenin and luteolin [21]. Interestingly, the energy values of our focused docking indicate that flavone, luteolin, and apigenin may interact with both HDAC1 and HDAC2 better than vorinostat. The differences in energy values are evident in both cases, but more relevant with HDAC1. The low values of energy obtained in our simulations strongly support the possibility that flavone, luteolin, and apigenin may interact with HDAC1 and HDAC2, thus confirming the experimental evidence [9,13,14,22].

### 3.3. Molecular Interactions of Flavones with HDAC1 and HDAC2

**Figure 3:**
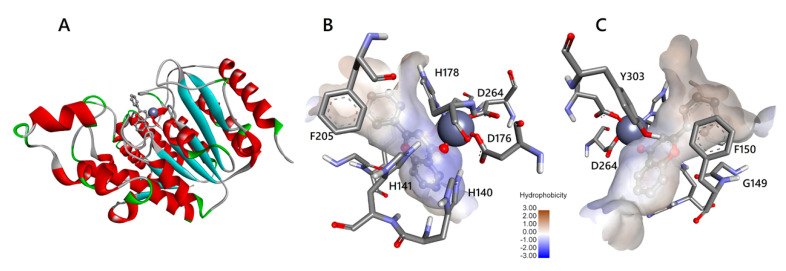
Interaction of flavone with HDAC1. (**A**) HDAC1 (ribbon with red helices and cyan strands) with flavone (grey molecule, in a ball and stick representation) in the interaction resulting from docking simulation. Zinc ion is represented as a sphere. (**B**) flavone (ball and stick) in the catalytic channel (surface coloured by hydrophobicity scale) of HDAC1. Amino acids (sticks) involved in the interaction with flavone are labelled with a one-letter code and number in the sequence. Zinc ion, represented as a sphere, is coordinated with the carbonyl oxygen atom of flavone ring C and D176, H178, D264. Atoms are coloured in grey (Carbon), red (Oxygen), white (Hydrogen), and blue (Nitrogen). (**C**) the opposite view of panel B shows the position of amino acids not visible in the other panel. Images are generated by DiscoveryStudio4.5.

**Figure 4:**
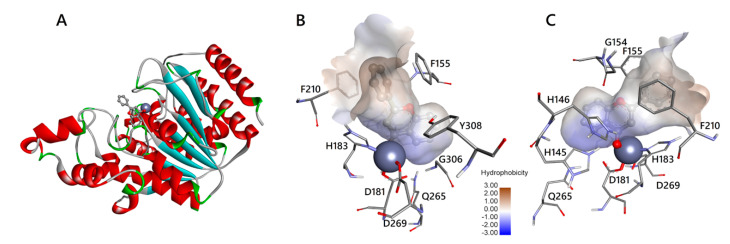
Interaction of flavone with HDAC2. (**A**) HDAC2 with flavone in the interaction with zinc ion resulting from docking simulation. Colours and representations are as in Figure 3. (**B**) flavone in the catalytic channel of HDAC2. Amino acids are labelled as in Figure 3. Zinc ion, represented as a sphere, is coordinated by the carbonyl oxygen of flavone ring C and D181, H183, D269. Atoms and bonds are coloured and represented as in Figure 3. (**C**) the opposite view of panel B shows the position of amino acids not visible in the other panel. Images are generated by DiscoveryStudio4.5.

Bontempo et al. (2007) [9] demonstrated that the anti-cancer pharmacological potential of the *Feijoa* fruits is due to the secondary metabolite flavone, which showed to inhibit HDAC1, thus hyper-acetylating histones and non-histone targets in leukaemia cell lines. To investigate the details of molecular interactions of flavone with HDAC1 and HDAC2, as also suggested by the energy values of our in silico simulations, we analysed the interactions of flavone in the active site binding pockets of HDAC1 and HDAC2 (see Figure 3 and Figure 4, respectively). A schematic 2D view is also reported in Supplementary Materials (Figures S5 and S6). Schematic 2D images for apigenin and luteolin are under Supplementary Figures S7–S8 and S9–S10, respectively. Apigenin and luteolin present additional hydroxyl groups that make possible different binding modes (not shown).

Overall, the three flavones are able to bind to the HDACs by completely inserting their aromatic rings into the active site pocket with multiple contacts to the tubular channel. Similar to the subclass benzamides of HDAC inhibitors, flavones are located in the active site pocket, more in depth than vorinostat belonging to the inhibitor’s subclass hydroxamic acids [23]. Indeed, as initially supposed by Bontempo et al. (2007) [9], flavone occupies in depth the catalytic channel of HDACs, binding the cofactor Zn^2+^ (Figure 3 and Figure 4 and Supplementary Video S1). HDACs deacetylate the ε-N-acetyl lysines of histones and non-histone targets in the active site channel of binding pocket about 11 Å deep, where the cofactor Zn^2+^ ion is located, by activating bound water for nucleophilic addition and subsequent hydrolysis [23,24]. The alkoxide zinc tetrahedral intermediate, stabilized by enzyme residues, releases the acetate and the lysine residue of target protein as reaction products. The tubular internal cavity about 14 Å deep is located below the active site and has been suggested to be an exit way for the acetate [23]. The first crystallographic structures of the human HDACs complexed with inhibitors support the mechanism of action of HDACi through binding to active site channels and steric hindrance preventing substrate hydrolysis [25]. In this regard, Bontempo et al. (2007) [9] hypothesized that flavone (and its derivatives) may similarly interact with the active site of HDAC occupying the same channel. According to the biochemical and structural characteristics of HDAC enzymes, a prototypical HDACi can be structurally subdivided into three functional regions: a polar tail (such as hydroxamic acid) chelates the catalytic Zn^2+^ ion located deep into the active site tunnel; a hydrophobic cap (aromatic group) is responsible for molecular recognition and selectivity of HDAC type closing the active site gate by interaction with the amino acid residues of the binding pocket rim; lastly, a central linker region places the two functional groups at the correct distance and interacts with the residues of the tunnel wall. Flavones do not structurally resemble canonical HDACi, lacking a true cap, but they can be considered to have a zinc binding group (carbonyl oxygen of ring C) and a linker (ring B). Nowadays, however, the classic inhibitor structure is questionable, because in most cases HDACis are profoundly different and cannot be dissected into the canonical zinc binding-linker-cap structure [26].

The amino acids of HDACs involved in the interaction with flavone, apigenin, luteolin, and vorinostat, are reported in Supplementary Table S3. First of all, we note that all residues involved at least in one of the interactions are conserved in HDAC1 and HDAC2. It is relevant to note that all ligands interact with the cofactor Zn^2+^ of both HDAC proteins. In fact, as already shown in Figure 3 and Figure 4, the atom of carbonyl oxygen of flavone, belonging to the heterocyclic ring C, can coordinate with the active site Zn^2+^ ion. The carbonyl carbon C4 of flavone should mime the carbonyl carbon of the acetyl moiety in the acetylated lysine residue of the substrate. The aromatic configuration of flavone hinders the nucleophilic attack by a water molecule. The chelation of the zinc ion by flavone differs from the bidentate binding mode proposed for class I HDAC (HDAC1, 2, 3, 8), based on the different structure of vorinostat, which binds the Zn^2+^ ion with two oxygen atoms in the hydroxamic acid group [27]. Flavone binding is possibly strengthened by an aromatic interaction with Tyr303/308 in HDAC1/2. Previous findings on in silico triple mutants indicate that this tyrosine residue, when present together with two phenylalanines, is essential to determine the chelation mode with vorinostat [27]. Our simulations confirm that two conserved phenylalanine residues (Phe150/155 and Phe205/210 in HDAC1/2) are located around the gateway of the binding pocket. Their phenyl groups are orientated in parallel to bind the ring B of flavone with a stacking configuration in both HDACs (Figure 3 and Figure 4). As proposed by Wu et al. (2011) [27], this “sandwich-like” conformation, when the two phenylalanines bind the linker region of vorinostat, blocks the binding pocket entrance and prevents water molecules from entering the channel.

All ligands interact with Gly149/154 and Gly301/306 of HDAC1/2 by van der Waals forces. Although no evidence of a key role is reported for these two amino acids, we note that Gly149/154 is spatially located right at the entrance of the binding pocket between the two conserved phenylalanines (the aforementioned Phe150/155 and Phe205/210), contributing to making the channel wall take its shape. Therefore, the space made available by the absence of a side chain for this glycine is needed to create an appropriate entrance for the substrate, as for the inhibitor flavone. Gly301/306 is located in a stretch of glycine residues (positions 299–302/304–307) of a loop region that, in the same way, generates an internal cavity adjacent to the binding pocket (data not shown).

Moreover, in all ligand-protein complexes with HDAC1/2, there is an interaction with Asp176/181 and His178/183. These two amino acids play an important role, because the coordination of Zn^2+^ is given by Asp176/181, His178/183, and Asp264/269. The latter amino acid interacts with ligands only in some complexes.

The proposed catalytic mechanism for deacetylation assigns also an important role to His140/145, His141/146, and Tyr303/308 [5], in particular with the histidines contributing to the charge-relay system of the active site. It is worth noting that our results show that these amino acids are involved in the interaction with vorinostat as well as with flavones in both HDACs (Supplementary Table S3).

Finally, four residues of HDAC1, i.e., Met30, Leu139, Cys151, and Gly300, such as two residues in HDAC2, i.e., Asp104 and Gln265, are always involved in ligand–protein binding. Although no evidence of a key role is reported, based on their location, they could contribute to shaping the binding pocket.

The dietary flavones apigenin and luteolin showed profiles of interaction similar to that of flavone. Overall, the position of flavones (having direct interactions with amino acids involved in relevant activities) midway between the active site channel and the tubular cavity, and their direct binding with the catalytic Zn^2+^ ion are suitable to prevent the interaction of HDACs with acetylated lysine residues as a substrate.

## 4. Conclusions

The aim of our work was to verify whether the three flavones, which were the object of our study (flavone, apigenin, luteolin), already known for their inhibitory activity on HDACs, can physically interact with HDAC1 and 2 similar to the known inhibitor vorinostat. Therefore, we used docking simulations for checking the ability of these three molecules to occupy the same binding site. The similar or better binding energy values for flavones suggest that it is possible. In more details, flavones are able to interact with HDAC1 and HDAC2 with energies similar to the known inhibitor vorinostat by occupying the catalytic site and creating interactions with Zn^2+^ ion and amino acids in the binding pocket. The present study contributed to shedding light on the molecular basis of the pharmacological potential of flavones as naturally occurring and no canonical HDACi. These plant secondary metabolites could be an important weapon against several diseases by epigenetic therapy. In the future, new “smart” drugs to fight cancer could have a natural origin and, thanks to the knowledge of their action mechanism, present reduced adverse effects by exerting anti-cancer activities via epigenetic modulation on responsive cells, such as HDAC inhibition. We would experimentally verify these data to further understand the selectivity of the flavones for the inhibitor effects on the different HDACs. Our findings could stimulate further investigations on these inhibitors by in vitro and in vivo model systems and be a support for the development of more selective and potent therapeutic molecules.

## Figures and Tables

**Table 1:** Predicted energies of interaction from molecular docking simulations of HDAC1/2 with flavones as ligands and inhibitor vorinostat as a reference.

Protein–Ligand Complex	Free Energy of Interaction (Kcal/mol)	Explicit Interaction with Zn^2+^ Ion	Rigid/Flexible Docking	Notes
**Blind docking**				
HDAC1-flavone	−8.50	yes	Rigid	
HDAC1-luteolin	−7.09	yes	Rigid	
HDAC1-apigenin	−7.61	yes	Rigid	
HDAC1-vorinostat	−7.23	yes	Rigid	
HDAC2-flavone	−7.94	yes	Rigid	
HDAC2-luteolin	−6.84	no	Rigid	
HDAC2-apigenin	−8.98	yes	flexible	hydrated with 3 water molecules
HDAC2-vorinostat	−7.45	yes	Rigid	Hydrated
**Focused docking**				
HDAC1-flavone	−10.25	Yes	flexible	
HDAC1-luteolin	−9.41	Yes	flexible	
HDAC1-apigenin	−9.25	Yes	flexible	
HDAC1-vorinostat	−8.46	Yes	rigid	hydrated
HDAC2-flavone	−8.90	Yes	rigid	
HDAC2-luteolin	−9.26	Yes	flexible	
HDAC2-apigenin	−9.32	Yes	flexible	
HDAC2-vorinostat	−8.45	Yes	rigid	2 water molecules involved

## Supplementary Materials

The following are available online at https://www.mdpi.com/2227-9059/8/12/568/s1, Supplementary File S1 containing Tables S1–S3 and Figures S1–S10. Supplementary Video S1: 3D view of the catalytic site of HDAC1 with flavone.
