# Supplementary material for: Molecular Docking Simulations on Histone Deacetylases (HDAC)-1 and -2 to Investigate the Flavone Binding
Source: Biomedicines. 2020 Dec 4;8(12):568. doi: 10.3390/biomedicines8120568 (PMC7761979; doi:10.3390/biomedicines8120568)
Supplement: Supplementary file 1 [file biomedicines-08-00568-s001.zip › Supplementary-File-S1.pdf]

Supplementary Materials

Molecular docking simulations on histone deacetylases (HDAC)-1 and -2 to investigate the flavone binding

Bernardina Scafuri [1,†], Paola Bontempo [2,†], Lucia Altucci [2], Luigi De Masi [3,*] and Angelo Facchiano [4,*]

[1] Department of Chemistry and Biology "A. Zambelli", University of Salerno, Via Giovanni Paolo II, 132, 84084 Fisciano (Salerno), Italy; bscafuri@unisa.it

[2] University of Campania "Luigi Vanvitelli", Department of Precision Medicine, via L. De Crecchio 7, 80138 Naples, Italy; paola.bontempo@unicampania.it and lucia.altucci@unicampania.it

[3] National Research Council (CNR), Institute of Biosciences and BioResources (IBBR), via Università 133, 80055 Portici (Naples), Italy; luigi.demasi@ibbr.cnr.it

[4] National Research Council (CNR), Institute of Food Science (ISA), via Roma 64, 83100 Avellino, Italy; angelo.facchiano@isa.cnr.it

† These authors contributed equally to this research work.

* Correspondence: luigi.demasi@ibbr.cnr.it (L.D.M.) and angelo.facchiano@isa.cnr.it (A.F.)

Supplementary Tables

Table S1

Autodock settings for simulations performed as blind (A) and focused (B) docking.

All parameters not specified in this table or under Methods were left as default settings.

A. Blind docking

Complex	Docking protocol	Grid box (npts)	Spacing (Å)
HDAC1-flavone	rigid	106*112*126	0.513
HDAC1-luteolin	rigid	104*116*126	0.500
HDAC1-apigenin	rigid	118*124*126	0.480
HDAC1-vorinostat	rigid	106*112*126	0.513
HDAC2-flavone	rigid	118*108*126	0.508
HDAC2-luteolin	rigid	118*108*126	0.508
HDAC2-apigenin	rigid and hydrated	118*126*126	0.48
HDAC2-vorinostat	rigid	118*114*126	0.48

B. Focused docking

Complex	Docking protocol	Grid box (npts)	Spacing (Å)	Flexible residues
HDAC1-flavone	flexible	48*60*55	0.375	HIS140; HIS141; ASP176; ASP264; TYR303
HDAC1-luteolin	flexible	48*60*55	0.375	HIS140; HIS141; PHE150; ASP176; PHE 205; ASP264; TYR303
HDAC1-apigenin	flexible	48*60*55	0.375	HIS140; HIS141; ASP176; PHE205; ASP264; TYR30
HDAC1-vorinostat	rigid and hydrated	48*60*55	0.375	
HDAC2-flavone	Rigid	48*60*48	0.375	
HDAC2-apigenin	flexible	50*60*48	0.375	HIS145; HIS146; ASP181; PHE 210; ASP269; TYR308
HDAC2-luteolin	flexible	50*60*48	0.375	HIS145; HIS146; ASP181; PHE 210; ASP269; TYR308
HDAC2-vorinostat	Rigid	50*60*48	0.375	

Table S2

Analysis of the best complex conformations.

For each complex, the best cluster of conformations has been considered, and the best three energy values have been considered.

Complex	Number of conformation in the cluster	1st conf.	2nd conf.	3rd conf.	Mean energy	Dev.std
HDAC1-flavone	15	-10.29	-10.23	-9.86	-10.13	0.23
HDAC1-luteolin	9	-9.41	-9.26	-8.87	-9.18	0.28
HDAC1-apigenina	12	-9.25	-8.34	-7.73	-8.44	0.76
HDAC1-vorinostat	40	-8.46	-8.38	-8.21	-8.35	0.13
HDAC2-flavone	51	-8.90	-8.90	-8.90	-8.90	0.00 [1]
HDAC2-luteolin	6	-9.26	-9.13	-9.02	-9.14	0.12
HDAC2-apigenin	1	-9.32 [2]			-9.32	
HDAC2-vorinostat	63	-8.50	-8.36	-8.31	-8.39	0.10

[1] Standard deviation value obtained for HDAC2-flavone complex is equal to zero as a consequence of the same energy value observed for the first three conformations.

[2] For HDAC2-luteolin docking simulation, the best conformation obtained has not been clustered with other complex conformations.

Table S3

Summary of interactions occurring for each tested ligand with HDAC1 and HDAC2.

**HDACs: residue**	**Interaction with**			
	**flavone**	**vorinostat**	**apigenin**	**luteolin**
HDAC1: Zn^{2+}	X	X	X	X
HDAC2: Zn^{2+}	X	X	X	X
HDAC1: TYR24				X
HDAC2: TYR29				
HDAC1: HIS29				
HDAC2: HIS33		X		
HDAC1: PRO29				
HDAC2: PRO34		X		
HDAC1: MET30	X	X	X	X
HDAC2: MET35	X			
HDAC1: ASP99			X	X
HDAC2: ASP104	X	X	X	X
HDAC1: GLY138		X		
HDAC2: GLY143			X	
HDAC1: LEU139	X	X	X	X
HDAC2: LEU144	X		X	
HDAC1: HIS140	X	X	X	X
HDAC2: HIS145	X	X	X	X
HDAC1: HIS141	X	X	X	X
HDAC2: HIS146	X	X	X	X
HDAC1: GLY149	X	X	X	X
HDAC2: GLY154	X	X	X	X
HDAC1: PHE150	X	X	X	X
HDAC2: PHE155	X	X	X	X
HDAC1: CYS151	X	X	X	X
HDAC2: CYS156	X		X	X
HDAC1: ASP176	X	X	X	X
HDAC2: ASP181	X	X	X	X
HDAC1: HIS178	X	X	X	X
HDAC2: HIS183	X	X	X	X
HDAC1: TYR204		X		
HDAC2: TYR209				
HDAC1: PHE205	X	X	X	X
HDAC2: PHE210	X	X	X	X
HDAC1: PRO206				
HDAC2: PRO211			X	X
HDAC1: GLN260			X	X
HDAC2: GLN265	X	X	X	X
HDAC1: ASP264			X	
HDAC2: ASP269			X	X

HDAC1: LEU271	X	X	X	
HDAC2: LEU276	X	X	X	
HDAC1: GLY272		X		
HDAC2: GLY277				
HDAC1: GLY300	X	X	X	X
HDAC2: GLY305	X			X
HDAC1: GLY301	X	X	X	X
HDAC2: GLY306	X	X	X	X
HDAC1: GLY302				
HDAC2: GLY307			X	X
HDAC1: TYR303	X	X	X	X
HDAC2: TYR308	X	X	X	X

Supplementary Figures

```
HDAC1_HUMAN -MAQTQGTRRKVCYYYDGDVGNYYYGQGHPMKPHRIRMTHNLLLNYGLYRKMEIYRPHKA 59
HDAC2_HUMAN MAYSQGGGKKKVCYYYDGDIGNYYYGQGHPMKPHRIRMTHNLLLNYGLYRKMEIYRPHKA
               .  * ::*********:***************************************


HDAC1_HUMAN NAEEMTKYHSDDYIKFLRSIRPDNMSEYSKQMQRFNVGEDCPVFDGLFEFCQLSTGGSVA 119
HDAC2_HUMAN TAEEMTKYHSDEYIKFLRSIRPDNMSEYSKQMQRFNVGEDCPVFDGLFEFCQLSTGGSVA
            .**********:************************************************

                             140 141  149 150                     176 178
                                ||       ||                         | |
HDAC1_HUMAN SAVKLNKQQTDIAVNWAGGLHHAKKSEASGFCYVNDIVLAILELLKYHQRVLYIDIDIHH 179
HDAC2_HUMAN GAVKLNRQQTDMAVNWAGGLHHAKKSEASGFCYVNDIVLAILELLKYHQRVLYIDIDIHH
            .*****:****:************************************************
                                ||       ||                         | |
                             145 146  154 155                     181 183

                                   205
                                    |
HDAC1_HUMAN GDGVEEAFYTTDRVMTVSFHKYGEYFPGTGDLRDIGAGKGKYYAVNYPLRDGIDDESYEA 239
HDAC2_HUMAN GDGVEEAFYTTDRVMTVSFHKYGEYFPGTGDLRDIGAGKGKYYAVNFPMRDGIDDESYGQ
            **********************************************:*:*********
                                    |
                                   210

HDAC1_HUMAN IFKPVMSKVMEMFQPSAVVLQCGSDSLSGDRLGCFNLTIKGHAKCVEFVKSFNLPMLMLG 299
HDAC2_HUMAN IFKPIISKVMEMYQPSAVVLQCGADSLSGDRLGCFNLTVKGHAKCVEVVKTFNLPLLMLG
            ****::******:**********:**************:********.**:****:****

           301 303
             | |
HDAC1_HUMAN GGGYTIRNVARCWTYETAVALDTEIPNELPYNDYFEYFGPDFKLHISPSNMTNQNTNEYL 359
HDAC2_HUMAN GGGYTIRNVARCWTYETAVALDCEIPNELPYNDYFEYFGPDFKLHISPSNMTNQNTPEYM
            ********************** ********************************* **:
             | |
           306 308

HDAC1_HUMAN EKIKQRLFENLRMLPHAPGVQMQAIPEDAIPEESGDEDEDDPDKRISICSSDKRIACEEE 419
HDAC2_HUMAN EKIKQRLFENLRMLPHAPGVQMQAIPEDAVHEDSGDEDGEDPDKRISIRASDKRIACDEE
            *****************************: *:***** :******** :*******:**


HDAC1_HUMAN FSDSEEEGEGGRKNSSNFKKAKRVKTEDEKEKDPE-EKKEVTEEEKTKEE---KPEAKGV 475
HDAC2_HUMAN FSDSEDEGEGGRRNVADHKKGAKKARIEEDKKETEDKKTDVKEEDKSKDNSGEKTDTKGT
            *****:******:* ::.**. :    :*.:*: * :*.:*.**:*:*::   * ::**.


HDAC1_HUMAN KEEVKLA- 482
HDAC2_HUMAN KSEQLSNP 488
            *.*
```

Figure S1. Alignment of HDAC1 and HDAC2 sequences. Amino acids with numbers are in evidence for their interaction with ligands as described in the Results section. Numbers for HDAC2 are shifted in comparison to HDAC1 as we reported the numbers from PDB files.

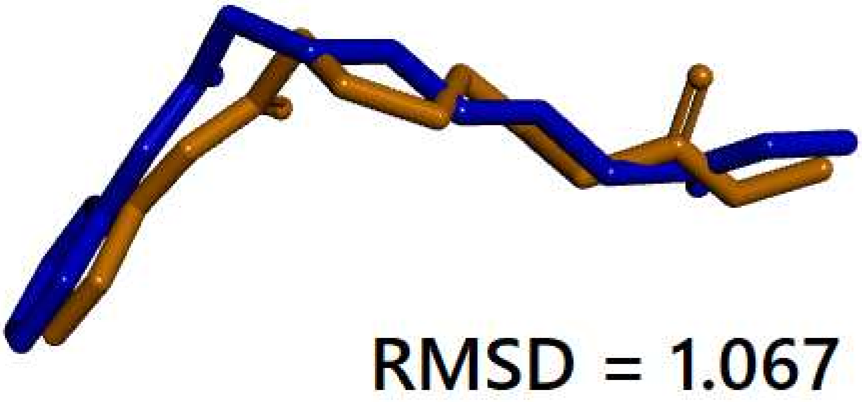

Figure S2. Superimposition of vorinostat molecules from the experimental complex and the redocked complex.

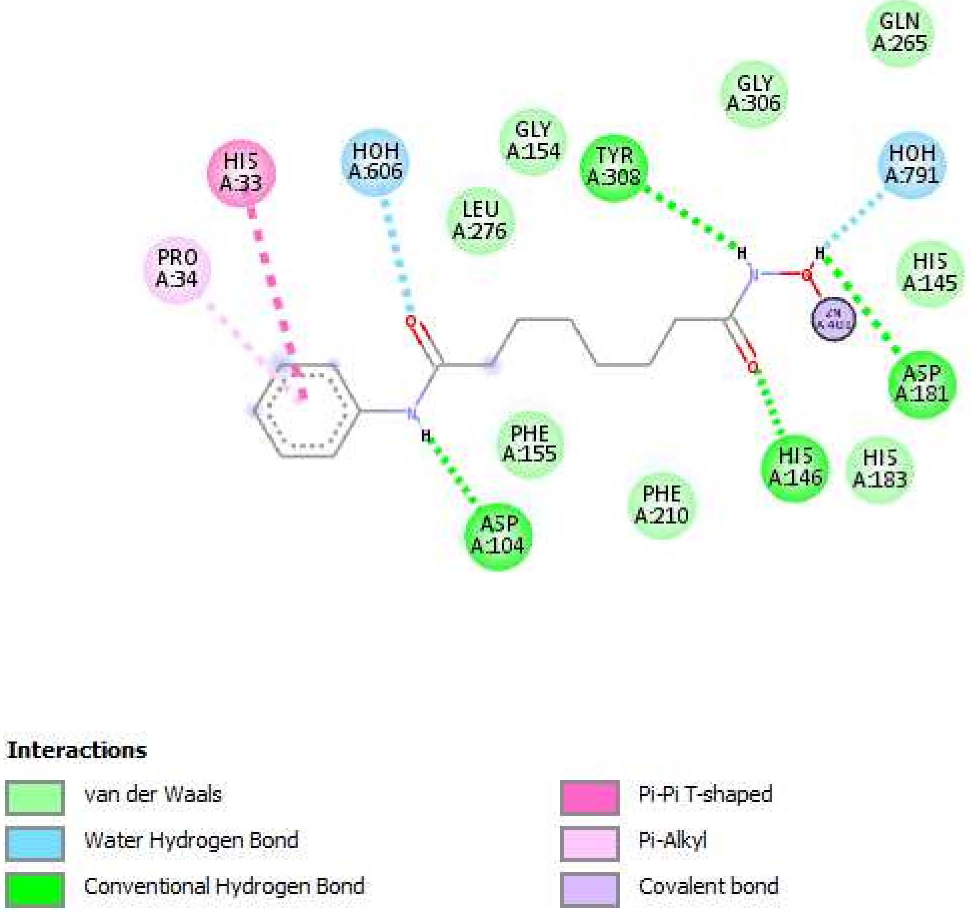

Figure S3. 2D diagram of interaction between HDAC2 and vorinostat. Image created with DiscoveryStudio4.5.

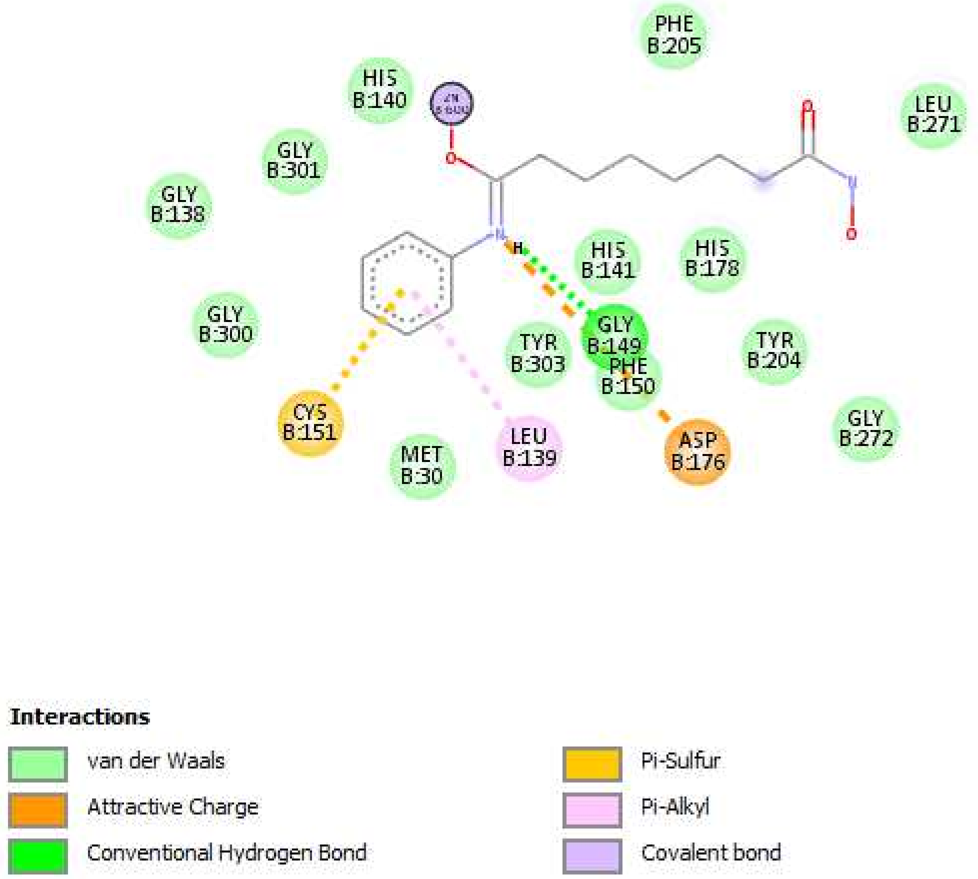

Figure S4. 2D diagram of interaction between HDAC1 and vorinostat. Image created with DiscoveryStudio4.5.

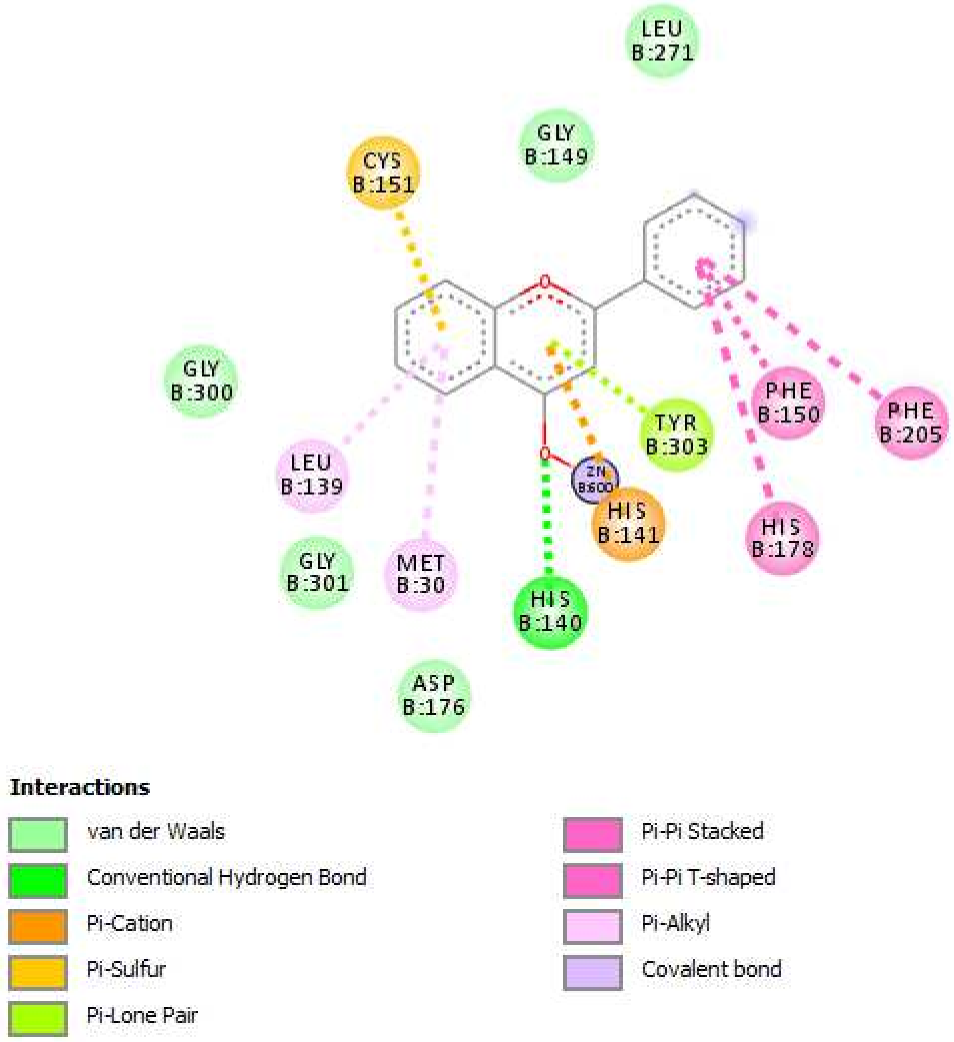

Figure S5. 2D diagram of interaction between HDAC1 and flavone. Image created with DiscoveryStudio4.5.

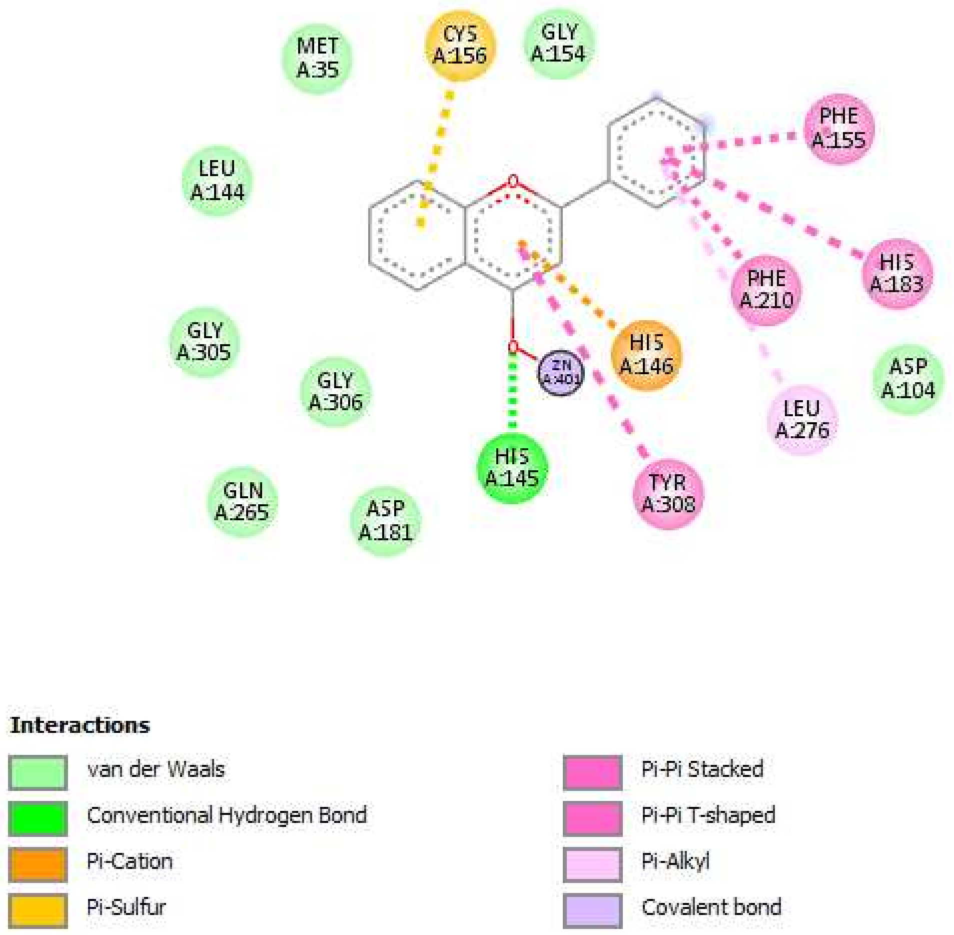

Figure S6. 2D diagram of interaction between HDAC2 and flavone. Image created with DiscoveryStudio4.5.

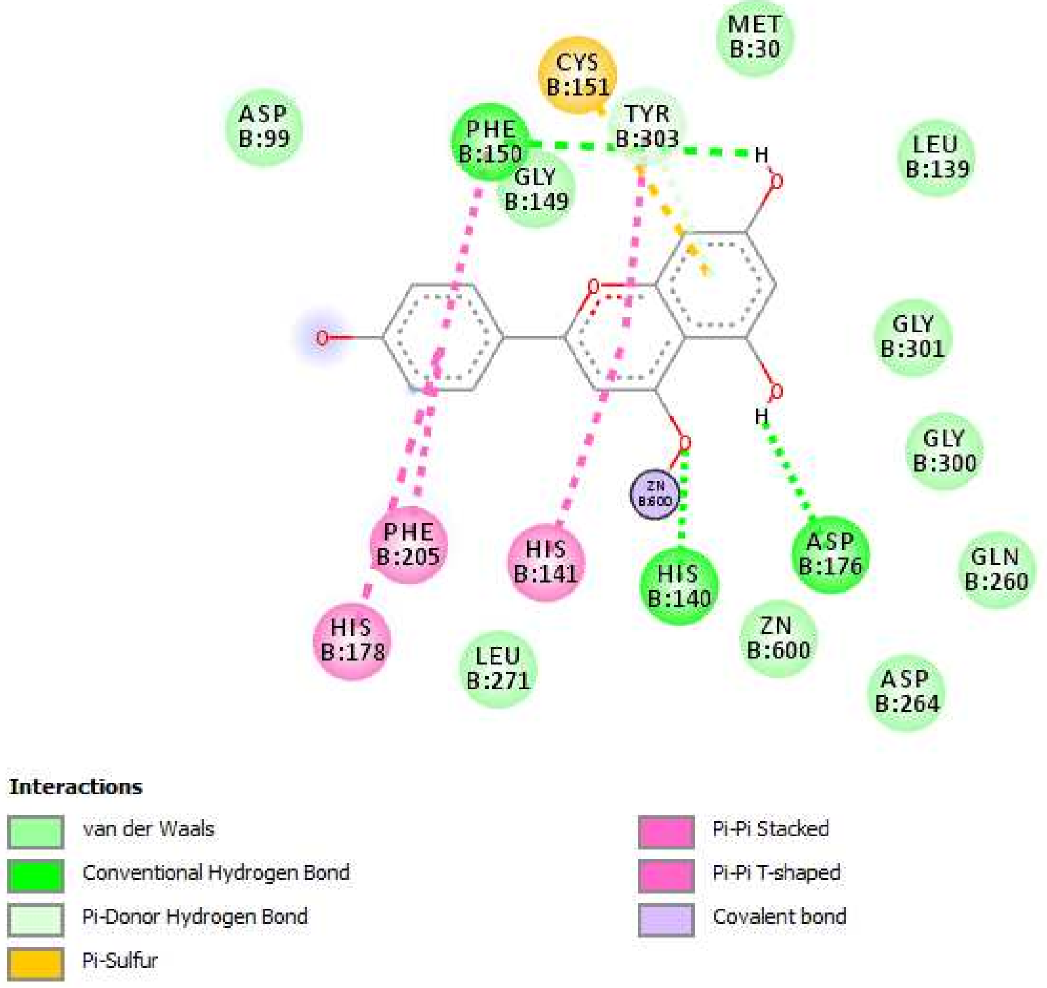

Figure S7. 2D diagram of interaction between HDAC1 and apigenin. Image created with DiscoveryStudio4.5.

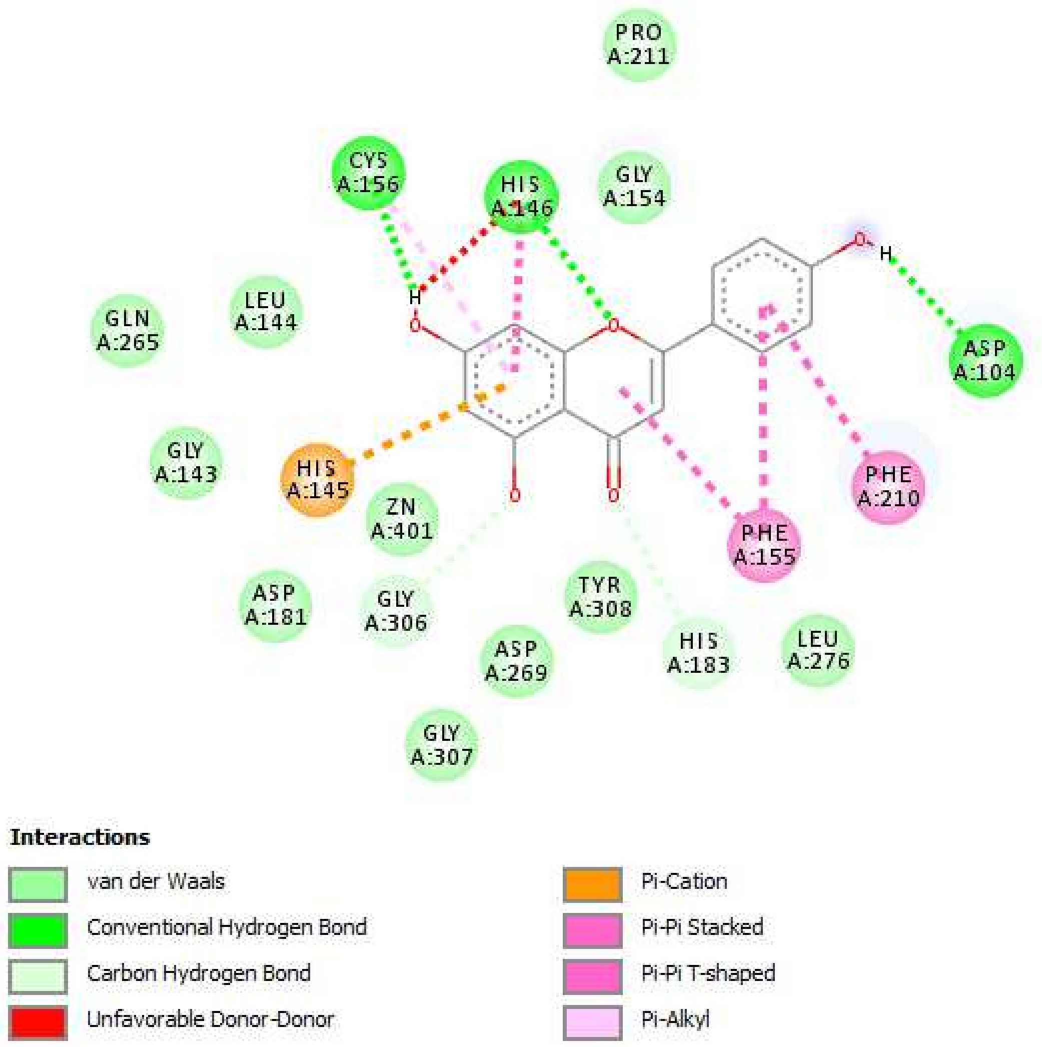

Figure S8. 2D diagram of interaction between HDAC2 and apigenin. Image created with DiscoveryStudio4.5.

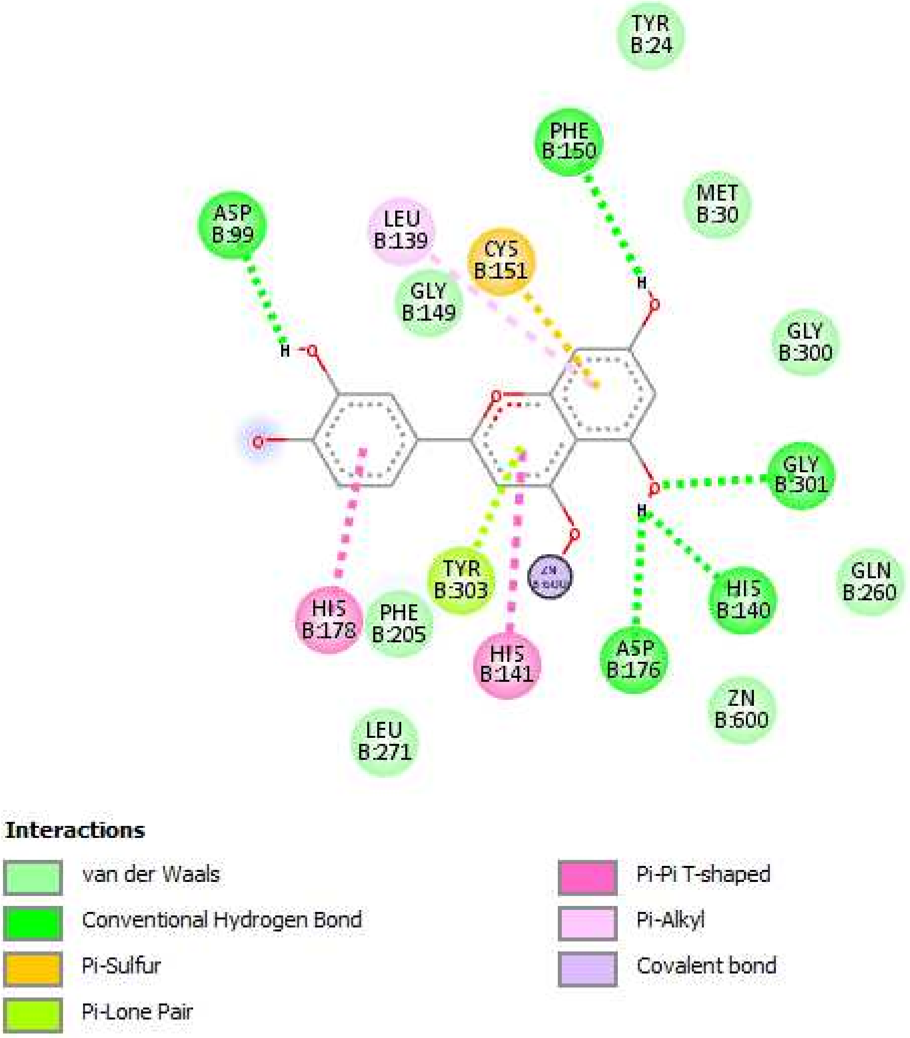

Figure S9. 2D diagram of interaction between HDAC1 and luteolin. Image created with DiscoveryStudio4.5.

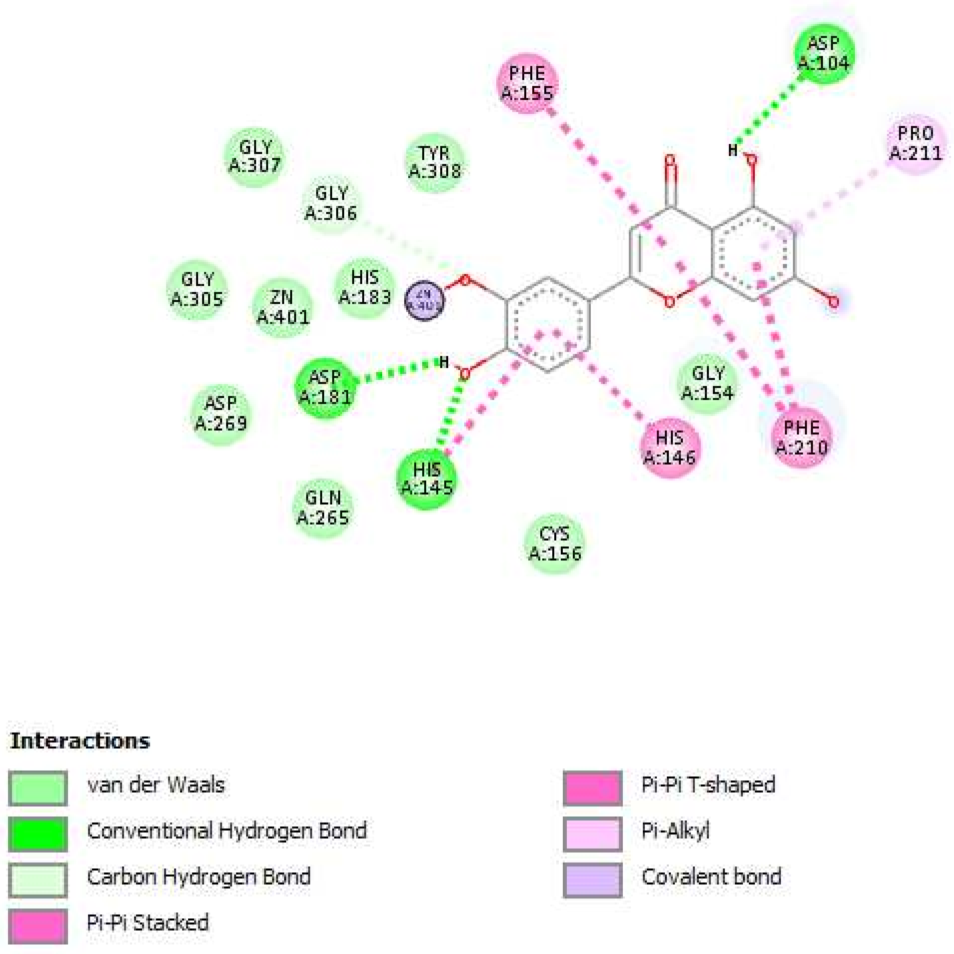

Figure S10. 2D diagram of interaction between HDAC2 and luteolin. Image created with DiscoveryStudio4.5.
